# (Methoxo-κ*O*)oxidobis(quinolin-8-olato-κ^2^ *N*,*O*)vanadium(V)

**DOI:** 10.1107/S1600536809031560

**Published:** 2009-08-15

**Authors:** Zhenghua Guo, Lianzhi Li, Chengyuan Wang, Tao Xu, Jinghong Li

**Affiliations:** a School of Chemistry and Chemical Engineering, Liaocheng University, Shandong 252059, People’s Republic of China; b Research Center of Medical Chemistry & Chemical Biology, Chongqing Technology and Business University, Chongqing 400067, People’s Republic of China

## Abstract

In the title complex, [V(C_9_H_6_NO)_2_(CH_3_O)O], the central V^V^ atom is coordinated by the O atoms from the oxido and methoxo ligands and the N and O atoms of two bis-chelating quinolin-8-olate ligands, forming a distorted octa­hedral environment. In the crystal structure, weak inter­molecular C—H⋯O hydrogen bonds connect mol­ecules into centrosymmetric dimers which are, in turn, linked by weak C—H⋯π inter­actions into chains along the *b* axis.

## Related literature

For the properties of vanadium compounds, see: Crans *et al.* (2004 ▶); Diego *et al.* (2003 ▶); Thompson & Orvig (2006 ▶). For the structures of oxidovandium complexes see: Hoshina *et al.* (1998 ▶); Otieno *et al.* (1996 ▶).
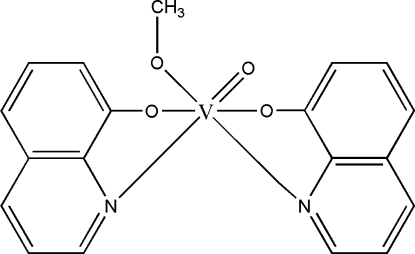


## Experimental

### 

#### Crystal data

[V(C_9_H_6_NO)_2_(CH_3_O)O]
*M* *_r_* = 386.27
Monoclinic, 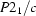

*a* = 14.0405 (16) Å
*b* = 8.0019 (1) Å
*c* = 15.5920 (18) Å
β = 110.560 (1)°
*V* = 1640.2 (3) Å^3^
*Z* = 4
Mo *K*α radiation
μ = 0.63 mm^−1^
*T* = 298 K
0.44 × 0.18 × 0.17 mm

#### Data collection

Bruker SMART 1000 CCD area-detector diffractometer
Absorption correction: multi-scan (*SADABS*; Sheldrick, 1996 ▶) *T*_min_ = 0.768, *T*_max_ = 0.900
7660 measured reflections
2893 independent reflections
1378 reflections with *I* > 2σ(*I*)
*R*_int_ = 0.102

#### Refinement

*R*[*F* ^2^ > 2σ(*F* ^2^)] = 0.072
*wR*(*F* ^2^) = 0.230
*S* = 1.00
2893 reflections
235 parameters
H-atom parameters constrained
Δρ_max_ = 0.81 e Å^−3^
Δρ_min_ = −0.69 e Å^−3^

### 

Data collection: *SMART* (Siemens, 1996 ▶); cell refinement: *SAINT* (Siemens, 1996 ▶); data reduction: *SAINT*; program(s) used to solve structure: *SHELXS97* (Sheldrick, 2008 ▶); program(s) used to refine structure: *SHELXL97* (Sheldrick, 2008 ▶); molecular graphics: *PLATON* (Spek, 2009 ▶); software used to prepare material for publication: *SHELXTL* (Sheldrick, 2008 ▶).

## Supplementary Material

Crystal structure: contains datablocks global, I. DOI: 10.1107/S1600536809031560/lh2870sup1.cif

Structure factors: contains datablocks I. DOI: 10.1107/S1600536809031560/lh2870Isup2.hkl

Additional supplementary materials:  crystallographic information; 3D view; checkCIF report

## Figures and Tables

**Table 1:** Selected bond angles (°)

O3—V1—O2	101.9 (2)
O3—V1—O1	91.4 (2)
O2—V1—O1	156.3 (2)
O3—V1—N1	164.3 (2)
O2—V1—N1	85.35 (19)
O1—V1—N1	77.40 (19)
O4—V1—N2	170.1 (2)

**Table 2:** Hydrogen-bond geometry (Å, °)

*D*—H⋯*A*	*D*—H	H⋯*A*	*D*⋯*A*	*D*—H⋯*A*
C9—H9⋯O4^i^	0.93	2.54	3.355 (8)	146
C19—H19*B*⋯*Cg*^ii^	0.96	2.84	3.520 (9)	128

Symmetry codes: (i) 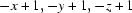; (ii) 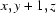. *Cg* is the centroid of the N2/C10–C14 ring.

**Fig. 1.**
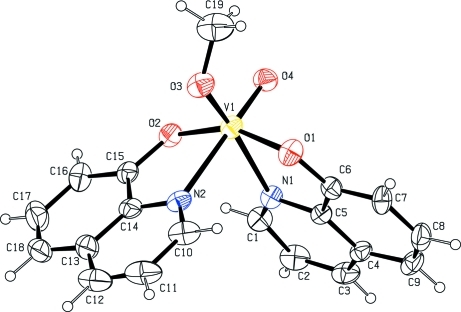
The molecular structure of the title compound, showing 30% probability displacement ellipsoids and the atom-numbering scheme.

**Fig. 2.**
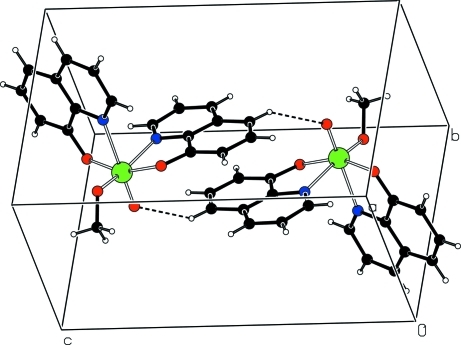
Part of the crystal structure with hydrogen bonds shown as dashed lines.

## supplementary crystallographic information

### Comment

Vanadium is a biologically essential trace element, encountered in metalloenzymes such as haloperoxidases or nitrogenases. Its coordination chemistry has received increasing attention due to the fact that vanadium compounds in various oxidation states have insulin-mimetic properties (Diego *et al.*, 2003; Crans *et al.*, 2004; Thompson & Orvig, 2006). We report here the synthesis and crystal structure of the title complex.

In the molecular structure (Fig.1.), the central V^V^ atom is six-coordinated by the O atoms of the oxo and methoxo ligands and the N atoms and O atoms of two 8-hydroxyquinolato ligands, forming a distorted octahedral environment (Table 1). The V=O bond distance is 1.602 (4) Å which is typical for oxovandium complexes (Hoshina *et al.*, 1998; Otieno *et al.*, 1996). The mean planes of the chelated rings defined by N1/C5—C6/O1/V1 and N2/C14—C15/O2/V1 form a dihedral angle of 82.02 (18)°.

In the crystal structure, weak intermolecular C—H···O hydrogen bonds connect molecules into centrosymmetric dimers (Fig. 2) which are, in turn, linked by weak C—H···π interactions into chains along the b axis.

### Experimental

8-Hydroxyquinoline (1 mmol, 145.16 mg) was dissolved in hot methanol (10 ml) and added dropwise to a methanol solution (3 ml) of VOSO_4_.3H_2_O (1 mmol, 225.4 mg). The mixture was then stirred at 323 K for 4 h. The solution was held at room temperature for 15 days, whereupon brown needle crystals suitable for X-ray diffraction were obtained.

### Refinement

All H atoms were placed in geometrically calculated positions, with C—H = 0.93–0.96 Å, and allowed to ride on their respective parent atoms, with *U*_iso_(H) = 1.2*U*_eq_(C) or 1.5*U*_eq_(C_methyl_).

### Crystal data

**Table:**

[V(C_9_H_6_NO)_2_(CH_3_O)O]	*F*(000) = 792
*M**_r_* = 386.27	*D*_x_ = 1.564 Mg m^−^^3^
Monoclinic, *P*2_1_/*c*	Mo *K*α radiation, λ = 0.71073 Å
Hall symbol: -P 2ybc	Cell parameters from 1311 reflections
*a* = 14.0405 (16) Å	θ = 2.7–25.3°
*b* = 8.0019 (1) Å	µ = 0.63 mm^−^^1^
*c* = 15.5920 (18) Å	*T* = 298 K
β = 110.560 (1)°	Needle, brown
*V* = 1640.2 (3) Å^3^	0.44 × 0.18 × 0.17 mm
*Z* = 4	

### Data collection

**Table:**

Bruker SMART 1000 CCD area-detector diffractometer	2893 independent reflections
Radiation source: fine-focus sealed tube	1378 reflections with *I* > 2σ(*I*)
graphite	*R*_int_ = 0.102
φ and ω scans	θ_max_ = 25.0°, θ_min_ = 1.6°
Absorption correction: multi-scan (*SADABS*; Sheldrick, 1996)	*h* = −16→16
*T*_min_ = 0.768, *T*_max_ = 0.900	*k* = −9→6
7660 measured reflections	*l* = −18→18

### Refinement

**Table:**

Refinement on *F*^2^	Primary atom site location: structure-invariant direct methods
Least-squares matrix: full	Secondary atom site location: difference Fourier map
*R*[*F*^2^ > 2σ(*F*^2^)] = 0.072	Hydrogen site location: inferred from neighbouring sites
*wR*(*F*^2^) = 0.230	H-atom parameters constrained
*S* = 1.00	*w* = 1/[σ^2^(*F*_o_^2^) + (0.1126*P*)^2^] where *P* = (*F*_o_^2^ + 2*F*_c_^2^)/3
2893 reflections	(Δ/σ)_max_ < 0.001
235 parameters	Δρ_max_ = 0.81 e Å^−^^3^
0 restraints	Δρ_min_ = −0.69 e Å^−^^3^

### Special details

**Table:**

Geometry. All e.s.d.'s (except the e.s.d. in the dihedral angle between two l.s. planes) are estimated using the full covariance matrix. The cell e.s.d.'s are taken into account individually in the estimation of e.s.d.'s in distances, angles and torsion angles; correlations between e.s.d.'s in cell parameters are only used when they are defined by crystal symmetry. An approximate (isotropic) treatment of cell e.s.d.'s is used for estimating e.s.d.'s involving l.s. planes.
Refinement. Refinement of *F*^2^ against ALL reflections. The weighted *R*-factor *wR* and goodness of fit *S* are based on *F*^2^, conventional *R*-factors *R* are based on *F*, with *F* set to zero for negative *F*^2^. The threshold expression of *F*^2^ > σ(*F*^2^) is used only for calculating *R*-factors(gt) *etc*. and is not relevant to the choice of reflections for refinement. *R*-factors based on *F*^2^ are statistically about twice as large as those based on *F*, and *R*-factors based on ALL data will be even larger.

### Fractional atomic coordinates and isotropic or equivalent isotropic displacement parameters (Å^2^)

**Table:**

	*x*	*y*	*z*	*U*_iso_*/*U*_eq_	
V1	0.26796 (9)	0.67329 (14)	0.24257 (8)	0.0458 (5)	
N1	0.3527 (4)	0.4493 (6)	0.3065 (4)	0.0403 (13)	
N2	0.1379 (4)	0.4855 (6)	0.1896 (4)	0.0446 (14)	
O1	0.2435 (3)	0.6607 (6)	0.3552 (3)	0.0536 (13)	
O2	0.2803 (3)	0.5922 (6)	0.1345 (3)	0.0479 (12)	
O3	0.1746 (3)	0.8276 (6)	0.2010 (4)	0.0592 (14)	
O4	0.3700 (3)	0.7827 (5)	0.2732 (3)	0.0522 (13)	
C1	0.4069 (5)	0.3432 (8)	0.2784 (5)	0.0504 (18)	
H1	0.4035	0.3502	0.2178	0.060*	
C2	0.4686 (5)	0.2221 (9)	0.3334 (6)	0.059 (2)	
H2	0.5043	0.1480	0.3100	0.071*	
C3	0.4762 (5)	0.2133 (8)	0.4233 (6)	0.058 (2)	
H3	0.5189	0.1345	0.4618	0.069*	
C4	0.4199 (5)	0.3227 (8)	0.4578 (5)	0.0426 (16)	
C5	0.3577 (5)	0.4370 (7)	0.3952 (4)	0.0370 (15)	
C6	0.2987 (5)	0.5570 (8)	0.4210 (5)	0.0427 (17)	
C7	0.3040 (5)	0.5595 (9)	0.5099 (4)	0.0522 (19)	
H7	0.2661	0.6366	0.5292	0.063*	
C8	0.3665 (6)	0.4458 (10)	0.5716 (5)	0.060 (2)	
H8	0.3695	0.4498	0.6321	0.072*	
C9	0.4238 (5)	0.3288 (9)	0.5485 (5)	0.056 (2)	
H9	0.4646	0.2549	0.5921	0.067*	
C10	0.0644 (5)	0.4382 (8)	0.2193 (6)	0.061 (2)	
H10	0.0650	0.4782	0.2755	0.073*	
C11	−0.0131 (6)	0.3317 (10)	0.1701 (8)	0.078 (3)	
H11	−0.0625	0.3005	0.1940	0.094*	
C12	−0.0177 (6)	0.2729 (10)	0.0881 (8)	0.081 (3)	
H12	−0.0704	0.2023	0.0550	0.097*	
C13	0.0588 (6)	0.3196 (9)	0.0526 (6)	0.061 (2)	
C14	0.1326 (5)	0.4281 (8)	0.1072 (5)	0.0467 (18)	
C15	0.2132 (5)	0.4850 (8)	0.0794 (5)	0.0440 (17)	
C16	0.2162 (6)	0.4307 (9)	−0.0033 (5)	0.058 (2)	
H16	0.2679	0.4669	−0.0232	0.070*	
C17	0.1425 (8)	0.3222 (11)	−0.0570 (6)	0.078 (3)	
H17	0.1458	0.2859	−0.1125	0.094*	
C18	0.0657 (7)	0.2675 (9)	−0.0307 (7)	0.076 (3)	
H18	0.0172	0.1948	−0.0683	0.092*	
C19	0.1912 (6)	1.0013 (10)	0.2138 (7)	0.088 (3)	
H19A	0.1692	1.0385	0.2623	0.132*	
H19B	0.1534	1.0588	0.1582	0.132*	
H19C	0.2624	1.0246	0.2295	0.132*	

### Atomic displacement parameters (Å^2^)

**Table:**

	*U*^11^	*U*^22^	*U*^33^	*U*^12^	*U*^13^	*U*^23^
V1	0.0373 (7)	0.0496 (8)	0.0487 (8)	0.0070 (6)	0.0128 (6)	0.0060 (6)
N1	0.031 (3)	0.048 (3)	0.045 (4)	0.007 (3)	0.017 (3)	0.002 (3)
N2	0.034 (3)	0.044 (3)	0.057 (4)	0.010 (3)	0.018 (3)	0.014 (3)
O1	0.046 (3)	0.060 (3)	0.056 (3)	0.021 (2)	0.020 (3)	0.007 (3)
O2	0.035 (3)	0.058 (3)	0.050 (3)	−0.002 (2)	0.015 (2)	0.008 (2)
O3	0.044 (3)	0.060 (3)	0.072 (4)	0.012 (2)	0.018 (3)	0.014 (3)
O4	0.041 (3)	0.050 (3)	0.061 (3)	−0.003 (2)	0.012 (3)	0.004 (2)
C1	0.051 (4)	0.051 (4)	0.055 (5)	0.012 (4)	0.027 (4)	−0.001 (4)
C2	0.047 (5)	0.054 (5)	0.086 (7)	0.013 (4)	0.036 (5)	−0.001 (4)
C3	0.042 (4)	0.045 (4)	0.081 (6)	0.009 (3)	0.016 (4)	0.013 (4)
C4	0.033 (4)	0.045 (4)	0.048 (5)	0.001 (3)	0.010 (3)	0.012 (4)
C5	0.027 (3)	0.042 (4)	0.043 (4)	0.000 (3)	0.013 (3)	0.006 (3)
C6	0.028 (4)	0.049 (4)	0.048 (5)	−0.001 (3)	0.009 (3)	−0.002 (4)
C7	0.049 (5)	0.077 (5)	0.034 (4)	0.002 (4)	0.019 (4)	−0.005 (4)
C8	0.059 (5)	0.084 (6)	0.035 (4)	−0.012 (5)	0.014 (4)	0.001 (4)
C9	0.038 (4)	0.068 (5)	0.052 (5)	0.002 (4)	0.006 (4)	0.024 (4)
C10	0.040 (4)	0.055 (5)	0.098 (7)	0.010 (4)	0.037 (5)	0.013 (5)
C11	0.043 (5)	0.059 (6)	0.139 (10)	0.002 (4)	0.039 (6)	0.019 (6)
C12	0.033 (5)	0.050 (5)	0.135 (10)	0.000 (4)	−0.002 (6)	0.018 (6)
C13	0.046 (5)	0.053 (5)	0.064 (6)	0.004 (4)	−0.006 (4)	0.004 (4)
C14	0.039 (4)	0.036 (4)	0.057 (5)	0.009 (3)	0.006 (4)	0.010 (4)
C15	0.032 (4)	0.044 (4)	0.048 (5)	0.009 (3)	0.004 (4)	0.010 (4)
C16	0.056 (5)	0.070 (5)	0.047 (5)	0.018 (4)	0.017 (4)	−0.002 (4)
C17	0.089 (7)	0.069 (6)	0.059 (6)	0.013 (5)	0.004 (6)	−0.018 (5)
C18	0.071 (6)	0.043 (5)	0.077 (7)	0.002 (4)	−0.022 (5)	0.001 (5)
C19	0.061 (6)	0.072 (6)	0.135 (9)	0.015 (5)	0.040 (6)	0.016 (6)
C19	0.061 (6)	0.072 (6)	0.135 (9)	0.015 (5)	0.040 (6)	0.016 (6)

### Geometric parameters (Å, °)

**Table:**

V1—O4	1.602 (4)	C7—C8	1.390 (9)
V1—O3	1.752 (5)	C7—H7	0.9300
V1—O2	1.870 (5)	C8—C9	1.363 (9)
V1—O1	1.907 (5)	C8—H8	0.9300
V1—N1	2.188 (5)	C9—H9	0.9300
V1—N2	2.284 (6)	C10—C11	1.383 (11)
N1—C1	1.314 (7)	C10—H10	0.9300
N1—C5	1.364 (7)	C11—C12	1.341 (12)
N2—C10	1.326 (8)	C11—H11	0.9300
N2—C14	1.342 (8)	C12—C13	1.421 (12)
O1—C6	1.335 (7)	C12—H12	0.9300
O2—C15	1.339 (8)	C13—C14	1.390 (10)
O3—C19	1.412 (9)	C13—C18	1.400 (12)
C1—C2	1.380 (9)	C14—C15	1.422 (9)
C1—H1	0.9300	C15—C16	1.375 (9)
C2—C3	1.369 (10)	C16—C17	1.385 (11)
C2—H2	0.9300	C16—H16	0.9300
C3—C4	1.408 (9)	C17—C18	1.353 (12)
C3—H3	0.9300	C17—H17	0.9300
C4—C9	1.397 (9)	C18—H18	0.9300
C4—C5	1.397 (8)	C19—H19A	0.9600
C5—C6	1.416 (8)	C19—H19B	0.9600
C6—C7	1.362 (8)	C19—H19C	0.9600
			
O4—V1—O3	101.5 (2)	C6—C7—C8	119.4 (7)
O4—V1—O2	95.9 (2)	C6—C7—H7	120.3
O3—V1—O2	101.9 (2)	C8—C7—H7	120.3
O4—V1—O1	100.7 (2)	C9—C8—C7	123.7 (7)
O3—V1—O1	91.4 (2)	C9—C8—H8	118.1
O2—V1—O1	156.3 (2)	C7—C8—H8	118.1
O4—V1—N1	91.5 (2)	C8—C9—C4	118.3 (7)
O3—V1—N1	164.3 (2)	C8—C9—H9	120.8
O2—V1—N1	85.35 (19)	C4—C9—H9	120.8
O1—V1—N1	77.40 (19)	N2—C10—C11	122.6 (8)
O4—V1—N2	170.1 (2)	N2—C10—H10	118.7
O3—V1—N2	86.0 (2)	C11—C10—H10	118.7
O2—V1—N2	76.2 (2)	C12—C11—C10	120.8 (8)
O1—V1—N2	85.3 (2)	C12—C11—H11	119.6
N1—V1—N2	82.15 (19)	C10—C11—H11	119.6
C1—N1—C5	117.5 (6)	C11—C12—C13	119.2 (8)
C1—N1—V1	131.5 (5)	C11—C12—H12	120.4
C5—N1—V1	110.4 (4)	C13—C12—H12	120.4
C10—N2—C14	116.6 (6)	C14—C13—C18	118.4 (8)
C10—N2—V1	133.1 (5)	C14—C13—C12	115.4 (8)
C14—N2—V1	109.9 (4)	C18—C13—C12	126.2 (9)
C6—O1—V1	119.8 (4)	N2—C14—C13	125.4 (7)
C15—O2—V1	122.2 (4)	N2—C14—C15	113.6 (6)
C19—O3—V1	125.2 (5)	C13—C14—C15	121.0 (8)
N1—C1—C2	123.9 (7)	O2—C15—C16	123.9 (6)
N1—C1—H1	118.1	O2—C15—C14	117.8 (6)
C2—C1—H1	118.1	C16—C15—C14	118.3 (7)
C3—C2—C1	118.6 (6)	C15—C16—C17	120.1 (8)
C3—C2—H2	120.7	C15—C16—H16	119.9
C1—C2—H2	120.7	C17—C16—H16	119.9
C2—C3—C4	120.5 (7)	C18—C17—C16	121.7 (8)
C2—C3—H3	119.7	C18—C17—H17	119.1
C4—C3—H3	119.7	C16—C17—H17	119.1
C9—C4—C5	118.5 (6)	C17—C18—C13	120.4 (8)
C9—C4—C3	125.5 (7)	C17—C18—H18	119.8
C5—C4—C3	115.9 (6)	C13—C18—H18	119.8
N1—C5—C4	123.6 (6)	O3—C19—H19A	109.5
N1—C5—C6	114.3 (6)	O3—C19—H19B	109.5
C4—C5—C6	122.0 (6)	H19A—C19—H19B	109.5
O1—C6—C7	125.6 (6)	O3—C19—H19C	109.5
O1—C6—C5	116.3 (6)	H19A—C19—H19C	109.5
C7—C6—C5	118.1 (6)	H19B—C19—H19C	109.5
			
O4—V1—N1—C1	−80.2 (6)	C9—C4—C5—N1	175.8 (6)
O3—V1—N1—C1	133.7 (9)	C3—C4—C5—N1	−2.3 (9)
O2—V1—N1—C1	15.5 (6)	C9—C4—C5—C6	−0.4 (9)
O1—V1—N1—C1	179.1 (6)	C3—C4—C5—C6	−178.5 (6)
N2—V1—N1—C1	92.2 (6)	V1—O1—C6—C7	165.8 (5)
O4—V1—N1—C5	90.4 (4)	V1—O1—C6—C5	−12.9 (7)
O3—V1—N1—C5	−55.8 (10)	N1—C5—C6—O1	2.5 (8)
O2—V1—N1—C5	−173.9 (4)	C4—C5—C6—O1	179.1 (5)
O1—V1—N1—C5	−10.3 (4)	N1—C5—C6—C7	−176.3 (6)
N2—V1—N1—C5	−97.2 (4)	C4—C5—C6—C7	0.3 (9)
O3—V1—N2—C10	−74.5 (6)	O1—C6—C7—C8	−178.6 (6)
O2—V1—N2—C10	−177.7 (6)	C5—C6—C7—C8	0.1 (10)
O1—V1—N2—C10	17.3 (6)	C6—C7—C8—C9	−0.3 (11)
N1—V1—N2—C10	95.2 (6)	C7—C8—C9—C4	0.2 (11)
O3—V1—N2—C14	97.8 (4)	C5—C4—C9—C8	0.2 (10)
O2—V1—N2—C14	−5.4 (4)	C3—C4—C9—C8	178.1 (6)
O1—V1—N2—C14	−170.4 (4)	C14—N2—C10—C11	1.6 (10)
N1—V1—N2—C14	−92.5 (4)	V1—N2—C10—C11	173.5 (5)
O4—V1—O1—C6	−76.6 (5)	N2—C10—C11—C12	−1.0 (12)
O3—V1—O1—C6	−178.6 (5)	C10—C11—C12—C13	0.8 (12)
O2—V1—O1—C6	56.9 (7)	C11—C12—C13—C14	−1.3 (11)
N1—V1—O1—C6	12.6 (4)	C11—C12—C13—C18	179.0 (8)
N2—V1—O1—C6	95.6 (5)	C10—N2—C14—C13	−2.3 (9)
O4—V1—O2—C15	178.2 (5)	V1—N2—C14—C13	−176.0 (5)
O3—V1—O2—C15	−78.7 (5)	C10—N2—C14—C15	179.5 (5)
O1—V1—O2—C15	44.0 (7)	V1—N2—C14—C15	5.8 (6)
N1—V1—O2—C15	87.2 (5)	C18—C13—C14—N2	−178.1 (6)
N2—V1—O2—C15	4.1 (4)	C12—C13—C14—N2	2.1 (10)
O4—V1—O3—C19	−12.4 (7)	C18—C13—C14—C15	0.0 (10)
O2—V1—O3—C19	−111.0 (6)	C12—C13—C14—C15	−179.8 (6)
O1—V1—O3—C19	88.8 (6)	V1—O2—C15—C16	175.9 (5)
N1—V1—O3—C19	132.9 (8)	V1—O2—C15—C14	−2.3 (8)
N2—V1—O3—C19	174.0 (6)	N2—C14—C15—O2	−3.1 (8)
C5—N1—C1—C2	−0.5 (10)	C13—C14—C15—O2	178.6 (6)
V1—N1—C1—C2	169.5 (5)	N2—C14—C15—C16	178.6 (6)
N1—C1—C2—C3	−1.6 (11)	C13—C14—C15—C16	0.3 (10)
C1—C2—C3—C4	1.8 (11)	O2—C15—C16—C17	−178.7 (6)
C2—C3—C4—C9	−177.9 (7)	C14—C15—C16—C17	−0.5 (10)
C2—C3—C4—C5	0.0 (9)	C15—C16—C17—C18	0.4 (12)
C1—N1—C5—C4	2.5 (9)	C16—C17—C18—C13	−0.1 (13)
V1—N1—C5—C4	−169.6 (5)	C14—C13—C18—C17	−0.1 (11)
C1—N1—C5—C6	179.0 (6)	C12—C13—C18—C17	179.6 (8)
V1—N1—C5—C6	6.9 (6)		

### Hydrogen-bond geometry (Å, °)

**Table:**

*D*—H···*A*	*D*—H	H···*A*	*D*···*A*	*D*—H···*A*
C9—H9···O4^i^	0.93	2.54	3.355 (8)	146
C19—H19B···Cg^ii^	0.96	2.84	3.520 (9)	128

Symmetry codes: (i) −*x*+1, −*y*+1, −*z*+1; (ii) *x*, *y*+1, *z*.
